# Atypical pharmacology of schistosome TRPA1-like ion channels

**DOI:** 10.1371/journal.pntd.0006495

**Published:** 2018-05-10

**Authors:** Swarna Bais, Corbett T. Berry, Xiaohong Liu, Gordon Ruthel, Bruce D. Freedman, Robert M. Greenberg

**Affiliations:** Department of Pathobiology, School of Veterinary Medicine, University of Pennsylvania, Philadelphia, Pennsylvania, United States of America; McGill University, CANADA

## Abstract

Parasitic flatworms of the genus *Schistosoma* cause schistosomiasis, a neglected tropical disease estimated to affect over 200 million people worldwide. Praziquantel is the only antischistosomal currently available for treatment, and there is an urgent need for new therapeutics. Ion channels play key roles in physiology and are targets for many anthelmintics, yet only a few representatives have been characterized in any detail in schistosomes and other parasitic helminths. The transient receptor potential (TRP) channel superfamily comprises a diverse family of non-selective cation channels that play key roles in sensory transduction and a wide range of other functions. TRP channels fall into several subfamilies. Members of both the TRPA and TRPV subfamilies transduce nociceptive and inflammatory signals in mammals, and often also respond to chemical and thermal signals. We previously showed that although schistosomes contain no genes predicted to encode TRPV channels, TRPV1-selective activators such as capsaicin and resiniferatoxin elicit dramatic hyperactivity in adult worms and schistosomula. Surprisingly, this response requires expression of a *S*. *mansoni* TRPA1-like orthologue (SmTRPA). Here, we show that capsaicin induces a rise in intracellular Ca^2+^ in mammalian cells expressing either SmTRPA or a *S*. *haematobium* TRPA1 orthologue (ShTRPA). We also test SmTRPA and ShTRPA responses to various TRPV1 and TRPA1 modulators. Interestingly, in contrast to SmTRPA, ShTRPA is not activated by the TRPA1 activator AITC (allyl isothiocyanate), nor do *S*. *haematobium* adult worms respond to this compound, a potentially intriguing species difference. Notably, 4-hydroxynonenal (4-HNE), a host-derived, inflammatory product that directly activates mammalian TRPA1, also activates both SmTRPA and ShTRPA. Our results point to parasite TRPA1-like channels which exhibit atypical, mixed TRPA1/TRPV1-like pharmacology, and which may also function to transduce endogenous host signals.

## Author summary

Schistosomes are parasitic flatworms that infect hundreds of millions of people worldwide. They cause schistosomiasis, a disease with major consequences for human health and economic development. There is only a single drug available for treatment and control of this highly prevalent disease, and there is an urgent need for development of new treatments. TRP ion channels play key roles in sensory (and other) functions. One type of TRP channel, TRPV1, is activated by capsaicin, the active ingredient in hot peppers. However, schistosomes do not have any TRPV-like channels. Nonetheless, we previously showed that capsaicin and similar compounds induce dramatic hyperactivity in schistosomes, and that this response is abolished by suppressing expression of SmTRPA, a schistosome TRPA1-like channel. Mammalian TRPA1 channels are not sensitive to capsaicin. Here, we show that the SmTRPA channel itself responds to capsaicin, resulting in an influx of Ca^2+^ into cells. ShTRPA, a TRPA1-like channel from another schistosome, *S*. *haematobium*, is also sensitive to capsaicin. Thus, the pharmacology of schistosome TRPA1 channels apparently differs from that of host mammalian channels, a characteristic that could indicate mixed TRPA/TRPV functionality and might be exploitable for development of new antischistosomal drugs. Furthermore, we show that schistosome TRPA1-like channels are activated by host-derived compounds, perhaps indicating a mechanism by which the parasite can respond to host signals.

## Introduction

Schistosomiasis, caused by trematode flatworms of the genus *Schistosoma*, affects hundreds of millions worldwide [1, 2], resulting in chronic morbidity, compromised childhood development, and up to 200,000 deaths annually [3–5]. With no vaccine, treatment and control depend almost entirely on the single drug praziquantel (PZQ) [6–8], which, though effective against all species of human schistosomes, has significant limitations [9–11]. Reports of PZQ resistance, both in the field and experimentally-induced [10, 12, 13] further highlight the urgent need for new antischistosomals.

Ion channels, which underlie electrical excitability in cells, are critical components of the neuromuscular system and are validated targets for a wide variety of drugs, including many of the anthelmintics currently in use [14–16]. Nonetheless, the properties, physiological roles, and pharmacological sensitivities of only a small subset of schistosome ion channels have been analyzed in any detail. One largely unexplored group of schistosome (and other parasite) ion channels is the transient receptor potential (TRP) channel superfamily [17, 18]. TRP channels are non-selective cation channels that display a wide range of functions, often exhibiting polymodal activation mechanisms, with different, seemingly unrelated signals capable of opening an individual channel [19–21]. Though the full range of functions fulfilled by TRP channels remains incomplete, it is clear that they play critical roles in responses to all major classes of sensory stimuli, including light, sound, chemicals, temperature, and touch, in large part by regulating intracellular Ca^2+^ levels [22]. These and the other functions of TRP channels have made them particularly appealing as candidate therapeutic targets [23]. Interestingly, recent reports also indicate that PZQ interacts with mammalian TRP channels [24, 25].

TRP channels appear throughout the animal kingdom and have been classified into structurally-defined subfamilies [19, 26] termed TRPC, TRPM, TRPA, TRPV, TRPML, TRPP, TRPN, and TRPVL (specific to cnidarians [26]). Schistosome (and other parasitic platyhelminth) genomes exhibit a wide diversity of TRP channel genes, yet appear to lack any sequences encoding TRPV (or TRPN) homologs [17, 18, 27]; in contrast, free-living platyhelminths have TRPV-like channel genes [18]. Mammalian TRPV1, the best-studied member of the TRPV channel family, responds to nociceptive and inflammatory signals, temperature, and pH, and is activated by vanilloids such as capsaicin, the primary active ingredient in hot peppers [28–31]. Like TRPV channels, TRPA1 channels are gated by thermal, mechanical, and nociceptive stimuli [reviewed in 32]. Both mammalian TRPV1 and TRPA1 channels also respond directly or indirectly to several endogenous pro-inflammatory and other agents [33–37]. For example, 4-hydroxynonenal (4-HNE), an inflammatory α,β-unsaturated aldehyde produced in response to tissue injury, inflammation, or oxidative stress, directly activates mammalian TRPA1 [38]. Since adult schistosomes reside within the circulatory system of the mammalian host, it is possible that such host-derived signals impact the parasite, either by direct binding to, or modulation of, *S*. *mansoni* TRP channels.

We have shown that despite the absence of TRPV-like channel genes in schistosomes, *S*. *mansoni* adults respond to capsaicin and other selective TRPV1 activators with dramatic hyperactivity and rapid separation of male-female pairs [39]. Schistosomula, early intra-mammalian larval schistosomes, also exhibit hyperactivity when exposed to capsaicin, while free-swimming, infectious cercariae display disrupted and "confused" swimming behavior [39]. Capsaicin-induced adult hyperactivity exhibits TRPV1-like pharmacology, as it is eliminated by co-exposure to SB 366719, a TRPV1-selective inhibitor. Despite this TRPV1-like pharmacology, however, we found that adult capsaicin-elicited hyperactivity is eliminated by knockdown of SmTRPA, a *S*. *mansoni* TRPA1 orthologue. TRPA1 channels in other organisms act as chemosensors for several pungent irritants including mustard oil (allyl isothiocyanate; AITC), but not for capsaicin [40, 41]. Like capsaicin, AITC elicits hyperactivity in adult worms, and this response is also eliminated by knockdown of SmTRPA RNA.

Based on these findings, we hypothesized that SmTRPA has atypical, mixed TRPV1/TRPA1-like pharmacology [18, 39]. Here, we provide direct evidence to support that hypothesis, measuring Ca^2+^ signals to show that TRPV1 activators such as capsaicin induce Ca^2+^ influx in mammalian cells expressing either SmTRPA or its *S*. *haematobium* orthologue (ShTRPA). We furthermore show that, though their pharmacological sensitivities overlap, SmTRPA and ShTRPA nonetheless exhibit differences that could possibly signify distinctive responses to physiological signals. We also test an endogenous host inflammatory compound for interactions with SmTRPA and ShTRPA that might reveal a novel mechanism by which parasites respond to and exploit host-derived signals.

## Methods

### Ethics statement

This study was carried out in strict accordance with the recommendations in the Guide for the Care and Use of Laboratory Animals of the U.S. National Institutes of Health. Animal handling and experimental procedures were undertaken in compliance with the University of Pennsylvania's Institutional Animal Care and Use Committee (IACUC) guidelines (Animal Welfare Assurance Number: A3079-01). The IACUC approved these studies under protocol number 806056.

### Reagents

Capsaicin was from Cayman Chemical (Ann Arbor, MI), 4-hydroxynonenal (4-HNE) was from Abcam (Cambridge, MA), olvanil was from Tocris Bioscience (Minneapolis, MN), and allyl isothiocyanate (AITC) and serotonin were from Sigma-Aldrich (St. Louis, MO). Reagents were dissolved in dimethyl sulfoxide (DMSO; ATCC, Manassas, VA) for stock solutions and then diluted to an appropriate concentration in culture or recording media. All oligonucleotides were from Integrated DNA Technologies (IDT, Coralville, IA).

### Isolation of schistosomes

*Biomphalaria glabrata* snails infected with *S*. *mansoni* (NMRI strain, NR-21962), Swiss-Webster mice infected with *S*. *mansoni* (NMRI strain, NR-21963), and *S*. *haematobium* adults perfused from hamsters were provided by the NIAID Schistosomiasis Resource Center of the Biomedical Research Institute under NIH-NIAID contract HHSN2722010000051 for distribution through BEI Resources [42, 43]. *S*. *mansoni* adults were perfused at 6–7 weeks post infection from mice, as described [42, 44], and were maintained in Standard Schistosome Medium consisting of RPMI (ThermoFisher, Philadelphia, PA), plus 10% FBS (GemCell, Gemini Bio Products, West Sacramento, CA) and 100 U/ml penicillin/100 mg/ml streptomycin (Corning Life Sciences, Tewksbury, MA), at 37°C and 5% CO_2_.

### Sources of plasmids used for Ca^2+^ imaging studies

Codon optimization and gene synthesis of schistosome TRPA sequences was by Genscript (Piscataway, NJ). Codon optimization was based on protein sequence AMB20412.1 (NCBI) for *S*. *mansoni* SmTRPA [18, 39] and on protein sequence KGB35426.1 for *S*. *haematobium* ShTRPA, predicted from the genome database [45]. We subsequently subcloned codon-optimized SmTRPA into the pcDNA3.1/zeo^(+)^ expression vector. We first amplified the full-length, codon-optimized SmTRPA coding sequence by PCR, using high-fidelity Q5 DNA polymerase (NEB, Woburn, MA). PCR primers included 5' overlapping vector sequences and were designed for Gibson cloning [46] into the EcoRV site of pcDNA3.1/zeo^(+)^ using the NEBuilder Assembly Tool (NEB). The amplicon was gel purified and inserted into linearized pcDNA3.1/zeo^(+)^ using the Gibson Assembly Master Mix (NEB), as per manufacturer's instructions. For ShTRPA, the codon-optimized sequence was inserted directly into the EcoRV site of pcDNA3.1/zeo^(+)^ by Genscript. Insertion of Kozak consensus sequences [47] at the start sites of both coding regions changed the second amino acid of SmTRPA from lysine (K) to glutamic acid (E), and of ShTRPA from isoleucine (I) to valine (V). We confirmed all constructs by DNA sequencing (Eurofins Genomics, Louisville, KY). These schistosome TRPA clones were used to transfect cells for Ca^2+^ imaging and are designated pSmTRPA and pShTRPA. Although the coding sequence of ShTRPA was predicted from the *S*. *haematobium* genome [45], we have not assessed the accuracy of that prediction, as we did previously for SmTRPA [39].

Rat TRPV1 (prTRPV1) and rat TRPA1 (prTRPA1) clones in pcDNA3 were generously provided by David Julius (University of California San Francisco). The pGP-CMV-GCaMP6f plasmid, containing the Ca^2+^ sensor GCaMP6f [48], was from Addgene (Cambridge, MA). pcDNA3.1/zeo^(+)^ was from ThermoFisher.

### Exposure of schistosomes to pharmacological compounds and analysis of motility

Protocols were similar to those described previously [39]. Adult worms were tested in Standard Schistosome Medium at 37°C on a Tokai Hit (Shizuoka, Japan) thermoplate. Briefly, single adult parasites were each placed in individual wells of a 24-well plate for 15 min at 37°C to obtain a baseline level of activity using the imaging system and software described previously [39, 49]. Test compounds were then added to the medium to appropriate final concentrations, and motility measured again over the course of another 15 min. Each worm thus served as its own control. As our vehicle control, we used 0.1% DMSO. Serotonin (40 μM), which increases schistosome motility, and PZQ (500 nM), which paralyzes schistosomes, served as controls to confirm that the analysis system was measuring worm activity accurately. The imaging system uses a USB monochrome camera to acquire and save images from all wells simultaneously. We subsequently analyze these series of images using custom MATLAB software that measures absolute differences in pixel gray scale values between consecutive images within each region of interest. For each pair of successive frames, we summed changes in pixel intensity, yielding a measurement of the amount of motion within the region of interest between frames and a value corresponding to each worm's activity.

### Cell culture and transfection

CHO-K1 cells were obtained from American Type Culture Collection (ATCC). They were grown in Ham's F-12K (Kaighn's) medium (ThermoFisher) supplemented with 10% fetal bovine serum (GemCell, Gemini Bio Products), and 100 U/ml penicillin/100 mg/ml streptomycin (Corning Life Sciences) at 37°C and 5% CO_2_. For these experiments, cells ranged between passages 5 to 11. For transfection and subsequent Ca^2+^imaging, we plated 2.5x10^5^ cells 24 h prior to transfection onto a 35-mm glass-bottom dish (14 mm microwell, glass thickness 0; MatTek Corporation, Ashland, MA) that had been pre-coated with 0.1 mg/mL poly-d-lysine (Millipore Sigma, Billerica, MA). We co-transfected cells, using Lipofectamine 2000 reagent (ThermoFisher), with pGP-CMV-GCaMP6f plus: pSmTRPA; pShTRPA; pcDNA3.1/zeo^(+)^; prTRPV1; or prTRPA1.

### Ca^2+^ imaging

Ca^2+^ signals were measured in CHO-K1 cells with the genetically-encoded Ca^2+^ indicator GCaMP6f [48] at 48 h following transfection, using methods similar to those described by others [50]. Briefly, 48 h following transfection, cells were cultured in Dulbecco's phosphate-buffered saline (DPBS; Corning Life Sciences), pH 7.0, containing Ca^2+^ (0.9 mM) and Mg^2+^ (0.5 mM) on the stage of a Leica DMI4000 inverted fluorescence microscope (Leica Biosystems, Inc., Buffalo Grove, IL). In some instances, measurements were performed in Ca^2+^-free medium, which was formulated using Ca^2+^/Mg^2+^-free DPBS supplemented with 1 mM EGTA and 0.5 mM MgCl_2_. GCaMP6f signals were captured through a 20x objective (NA 0.70) with a 16-bit cooled EMCCD camera (C9100-13, Hamamatsu, Bridgewater, NJ), with appropriate excitation (450 ± 20 nm) and emission (500 ± 20 nm) filters, mounted on a Yokogawa CSU-X1 spinning disk confocal attachment (Yokogawa, Sugar Land, TX). The system is enclosed in a custom environmental chamber for temperature (37° C) and CO_2_ (5%) control. Tested compounds (and concentrations) were: capsaicin (5 μM, 10 μM, 30 μM, 70 μM); AITC (20 μM); olvanil (10 μM); 4-HNE (50 μM); and ionomycin (1 μM).

Cells expressing GCaMP6f were illuminated at 488nm and changes in fluorescence intensity of each cell were captured for 15 min at 2-second intervals. Baseline fluorescence was recorded from 1 min to 5 min prior to, and for ~11 min following, application of the compounds. Image acquisition and data analysis were performed using the Metamorph 7 imaging suite (Molecular Devices, Sunnyvale, CA). Each experimental condition was repeated at least 3 times. Fluorescence was quantified and normalized on a cell by cell basis (F_cell_-F_min_) where F_cell_ is the fluorescence in the region of interest following stimulation, and F_min_ is the baseline fluorescence before stimulation. The ratio of this value (ΔF) over initial fluorescence (F_o_) is plotted in the figures depicting change in fluorescence vs. time. We observed no change in fluorescence in CHO-K1 cells transfected only with pGP-CMV-GCaMP6f and exposed to compounds other than the Ca^2+^ ionophore ionomycin.

### Statistics

Data were analyzed with GraphPad Prism or Microsoft Excel, expressed as arithmetic means ± SEM, and tested for statistical significance using the statistical tests noted in the figure legends. Figures showing normalized change in fluorescence were analyzed and plotted using the R v.3.4.0 (ggplot2 package). In the drug response studies, each worm served as its own control, and we therefore compared means using paired t-tests (on the raw data, prior to normalization).

## Results

### The TRPV1 activator capsaicin triggers Ca^2+^ influx in cells expressing schistosome TRPA1-like channels

We previously showed [39] that capsaicin, a potent and selective activator/enhancer of mammalian TRPV1 channels [51], evokes hyperactivity in adult *S*. *mansoni* that is dependent on the expression of SmTRPA, a schistosome TRPA1-like gene [39].

**Fig 1:**
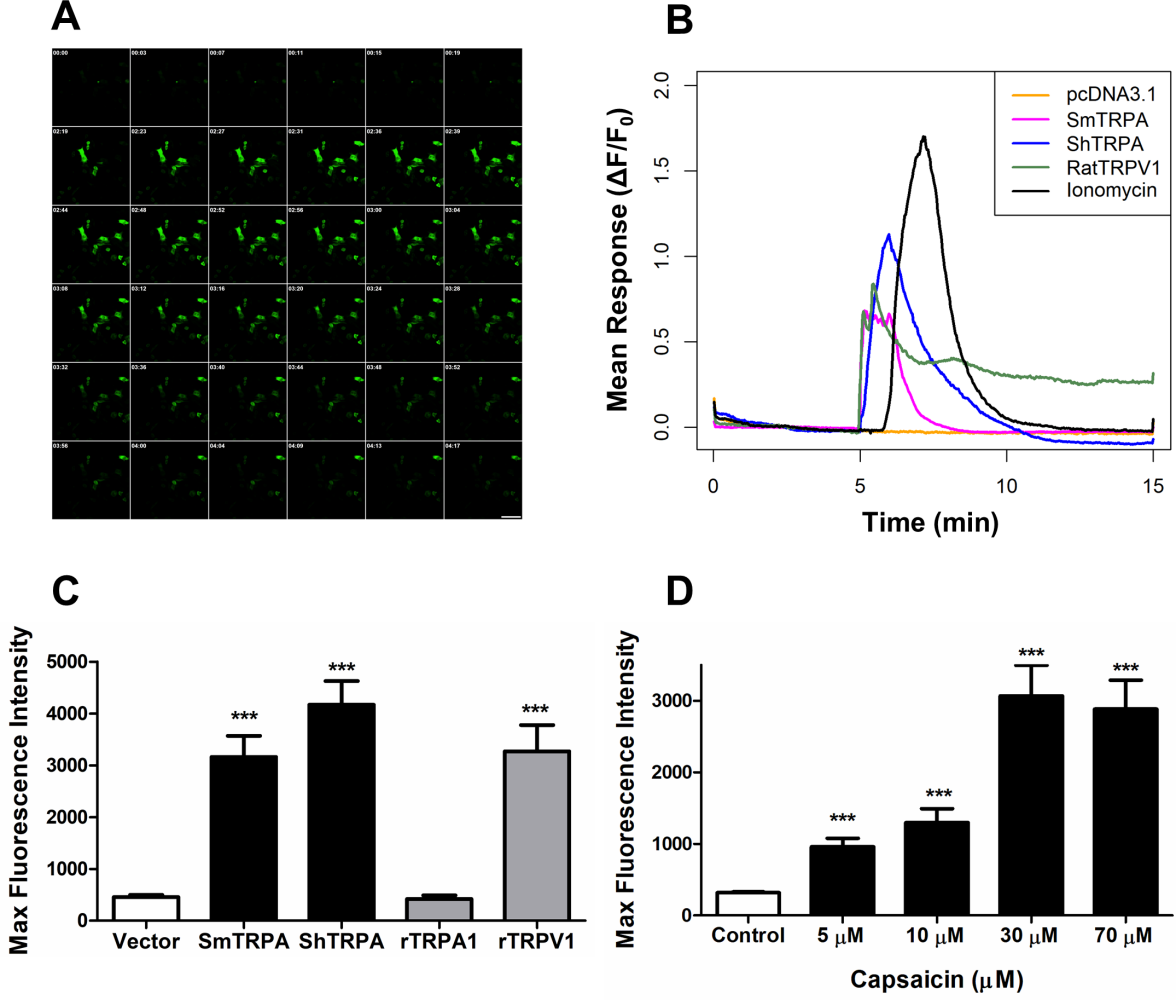
The TRPV1 activator capsaicin stimulates a significant Ca^2+^ influx in CHO cells expressing schistosome TRPA1-like channels. **A.** Montage showing the response of CHO cells co-transfected with SmTRPA and pGP-CMV-GCaMP6f to 10 μM capsaicin over time. Number in upper left corner of each picture represents elapsed time (in minutes:seconds). Capsaicin was applied at frame 6 (0:19). Calibration bar = 85 μm. **B.** Traces of averaged GCaMP6f fluorescence intensity change in response to 10 μM capsaicin in cells transfected with empty vector (pcDNA3.1/zeo^(+)^; orange), rat TRPV1 (green), SmTRPA (purple), or ShTRPA (blue). The black trace is a positive control indicating the averaged response of randomly selected cells (n = 90, 5 independent transfections) from each group to 1 μM ionomycin, a Ca^2+^ ionophore. Capsaicin was applied at 5 min. **C.** Normalized maximal GCaMP6f fluorescence intensity in response to 10 μM capsaicin in cells transfected with: empty vector (Vector; n = 62, 3 independent transfections); SmTRPA (n = 131 cells, 6 independent transfections); ShTRPA (n = 112, 6 independent transfections); rat TRPA1 (rTRPA1, n = 99, 3 independent transfections); and rat TRPV1 (rTRPV1, n = 41, 3 independent transfections). ***, P < 0.0001, unpaired, two-tailed t-test vs. empty vector data. Responses of cells transfected with SmTRPA or ShTRPA to capsaicin are also significantly higher (P <0.0001) than responses to DMSO (S1 Fig). **D.** Dose response of expressed SmTRPA to capsaicin. Normalized maximal GCaMP6f fluorescence in response to different concentrations of capsaicin is shown for CHO cells transfected with SmTRPA. Capsaicin concentrations tested are 5 μM (n = 53), 10 μM (n = 34), 30 μM (n = 121), 70 μM (n = 90). Control (n = 39) is 0.01% DMSO. In this experiment, all cells tested were from a single transfection, which likely accounts for the differences from the maximal fluorescence value at 10 μM shown in panel C, which represents results from multiple transfections.

To further address whether schistosome responses to capsaicin are mediated by parasite TRPA1-like channels, we expressed *S*. *mansoni* (SmTRPA) or *S*. *haematobium* (ShTRPA) TRPA1-like channels in CHO cells co-transfected with the genetically encoded Ca^2+^ indicator GCaMP6f [48], to assess the role of these channels in capsaicin-induced signaling. As shown in Fig 1A–1C, 10 μM capsaicin elicited a significant increase in cytoplasmic Ca^2+^ in CHO cells expressing either SmTRPA or ShTRPA. Similarly, and as expected, rat TRPV1-transfected cells also respond to capsaicin, though, unlike the relatively transient responses of cells expressing SmTRPA or ShTRPA, the Ca^2+^ signal in cells expressing rat TRPV1 appears to be sustained (compare green trace to blue and purple traces in Fig 1B). In contrast, there is no measurable response to 10 μM capsaicin in cells expressing rat TRPA1 or in those transfected with empty vector (pcDNA3.1/zeo^+^), nor was there any response to vehicle (0.01% DMSO) in cells expressing SmTRPA or ShTRPA (S1 Fig). Cells expressing SmTRPA respond to capsaicin concentrations of 5 μM and higher in a dose-dependent manner (Fig 1D). The capsaicin-induced signals in SmTRPA-expressing cells require extracellular Ca^2+^, as we observed no change in GCaMP6f fluorescence when cells were bathed in Ca^2+^-free medium (S2 Fig).

### Olvanil, another TRPV1 activator, triggers a rise in cytoplasmic Ca^2+^ in cells expressing schistosome TRPA1-like channels and increases motor activity in adult *S*. *mansoni*

**Fig 2:**
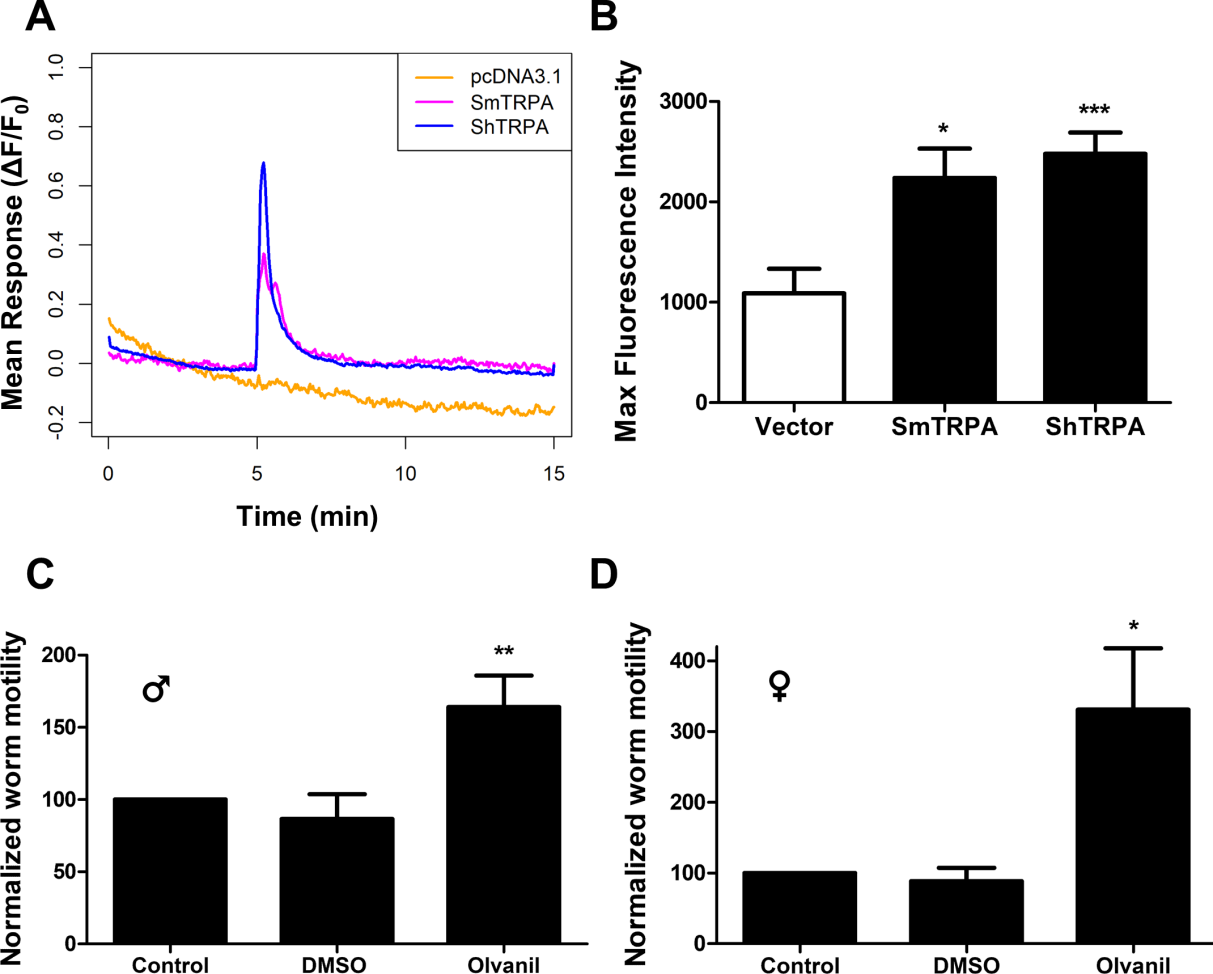
The TRPV1 activator olvanil stimulates an increase in intracellular Ca^2+^ in cells transfected with SmTRPA or ShTRPA and increases motor activity in adult *S*. *mansoni*. **A.** Traces of averaged GCaMP6f fluorescence intensity change in response to 10 μM olvanil in cells transfected with empty pcDNA3.1/zeo^(+)^ (orange), SmTRPA (purple), or ShTRPA (blue). Olvanil was applied at 5 min. **B.** Normalized maximal GCaMP6f fluorescence intensity in response to 10 μM olvanil in cells transfected with: empty vector (n = 67, 3 independent transfections); SmTRPA (n = 23, 4 independent transfections); ShTRPA (n = 177, 4 independent transfections). *, P < 0.05, ***, P < 0.0001, unpaired, two-tailed t-test vs. empty vector data. **C, D.** Measurement of motor activity in adult (~7 weeks post infection) *S*. *mansoni* males (C) or females (D) that were exposed in culture to 10 μM olvanil. Motility was assayed before and after addition of compound. "Control" contained no added compounds, and the "DMSO" sample contained 0.1% DMSO. Motility was analyzed as described [39], with each individual tested worm serving as its own control, and data normalized to Control response for each worm. *, P < 0.05, **, P < 0.01, paired, two-tailed t-test against Control, prior to normalization.

We next examined the response of cells expressing SmTRPA and ShTRPA to olvanil, a non-pungent synthetic analogue of capsaicin that potently activates TRPV1 [52]. As shown in Fig 2, olvanil induces a SmTRPA- or ShTRPA-dependent increase in intracellular Ca^2+^ (Fig 2A and 2B). Olvanil also evokes hyperactivity in adult *S*. *mansoni*, as both male and female schistosomes exposed to 10 μM olvanil exhibit significantly increased motility compared to controls (Fig 2C and 2D). In females (Fig 2D), the increased level of activity is comparable to that induced by 40 μM serotonin (S3 Fig), a classic stimulator of motor activity in schistosomes [53]. Males appear to be somewhat less responsive to olvanil than females (Fig 2C). As expected, 500 nM PZQ, used as a negative control, significantly reduces motility in both males and females (S3 Fig).

### The TRPA1 activator AITC triggers a rise in intracellular Ca^2+^ in cells expressing SmTRPA, but not ShTRPA

AITC is one of several pungent irritants that activate mammalian TRPA1 channels through reversible covalent modification of cysteine residues [54, 55]. At higher concentrations, AITC can also activate/sensitize TRPV1, via a mechanism independent of cysteine modification [56–58]. We previously showed that *S*. *mansoni* adults in culture respond to AITC with significant hyperactivity, and that this AITC sensitivity is eliminated upon knockdown of SmTRPA [39].

**Fig 3:**
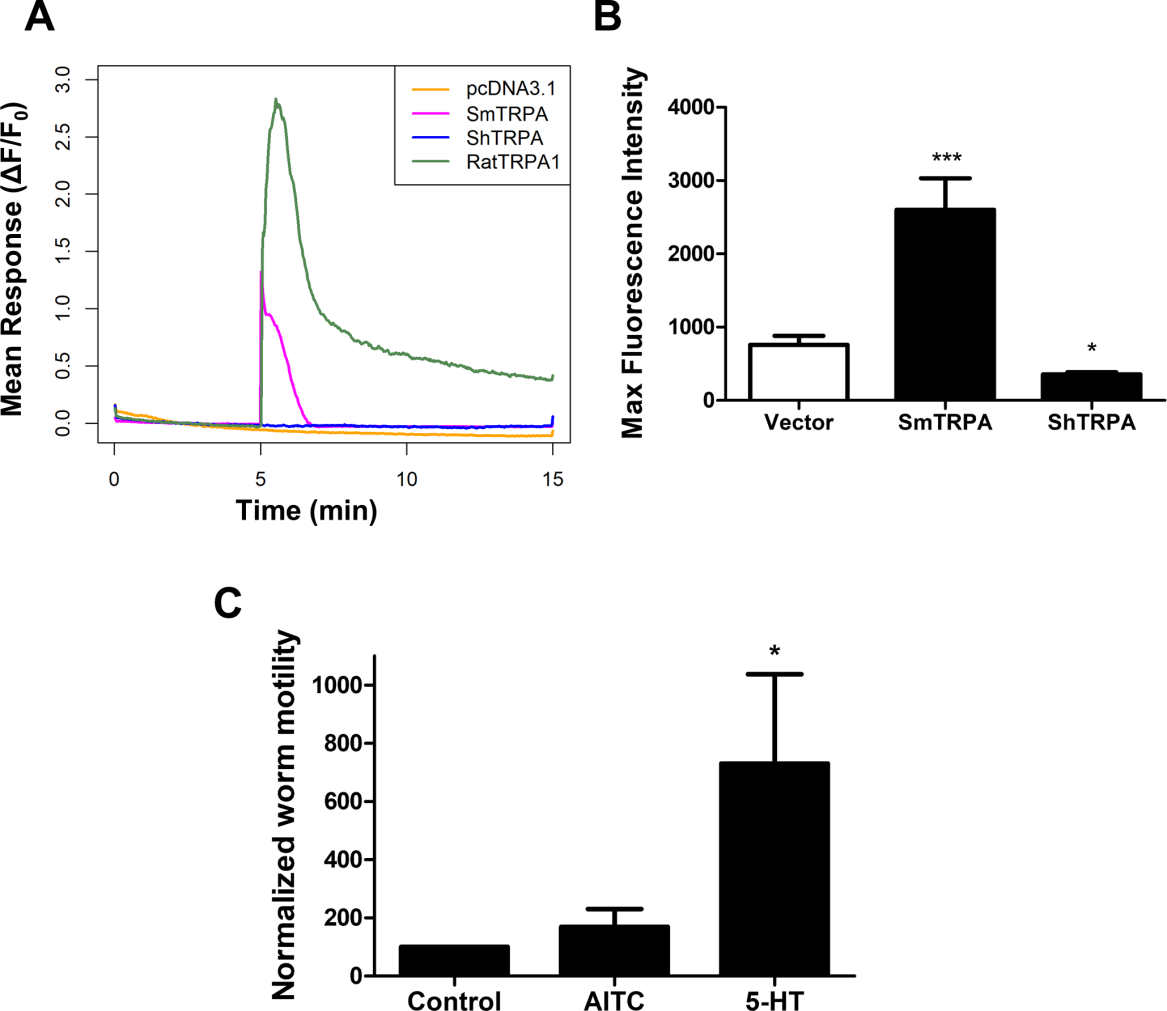
The TRPA1 activator AITC activates SmTRPA but not ShTRPA. **A.** Traces of averaged GCaMP6f fluorescence intensity change in cells transfected with empty vector (pcDNA3.1/zeo^(+)^; orange), rat TRPA1 (green), SmTRPA (purple), or ShTRPA (blue). **B.** Normalized maximal GCaMP6f fluorescence intensity in response to 20 μM AITC in cells transfected with empty vector (Vector, n = 149, 3 independent transfections); SmTRPA (n = 64, 4 independent transfections); or ShTRPA (n = 72, 4 independent transfections). *, P < 0.05, ***, P < 0.0001, unpaired two-tailed t-test vs. empty vector data. **C.** *S*. *haematobium* adult females do not exhibit hyperactivity in response to 60 μM AITC (n = 20). Activity was measured as described in Fig 2 and [39]. Data are normalized to Control response for each individual worm. As a positive control, we also tested 40 μM 5-HT, which does produce significant hyperactivity in *S*. *haematobium* females (n = 26). *, P < 0.05, paired, two-tailed t-test vs. Control, prior to normalization.

CHO cells expressing SmTRPA and GCaMP6f and treated with AITC (20 μM) exhibited a significant increase (3-4-fold; Fig 3) in GCaMP6f fluorescence (intracellular Ca^2+^ levels), comparable to that observed with capsaicin and olvanil (Figs 1 and 2). As expected, cells expressing rat TRPA1 also respond to AITC, though with a larger, >10-fold increase in GCaMP6f fluorescence, while cells expressing rat TRPV1 do not respond to AITC (Fig 3A, S4 Fig). Surprisingly, however, we observed no stimulatory effect of AITC at concentrations up to 100 μM on cells expressing *S*. *haematobium* ShTRPA. Indeed, as with the DMSO control (S1 Fig), AITC appears to produce a small, but significant, decrease in GCaMP6f fluorescence in cells transfected with ShTRPA. Consistent with these results, *S*. *haematobium* adults show no change in motor activity following exposure to AITC (Fig 3C), in contrast to *S*. *mansoni* adults, which exhibit significant AITC-elicited hyperactivity [39]. These results suggest a species difference in the pharmacological sensitivities of TRPA1-like channels in *S*. *mansoni* and *S*. *haematobium*, perhaps reflecting a divergence in responses to sensory or other signals transduced by this channel.

### 4-HNE, a host-derived TRPA1 activator, increases motor activity in *S*. *mansoni* and triggers a rise in intracellular Ca^2+^ in cells expressing SmTRPA or ShTRPA

**Fig 4:**
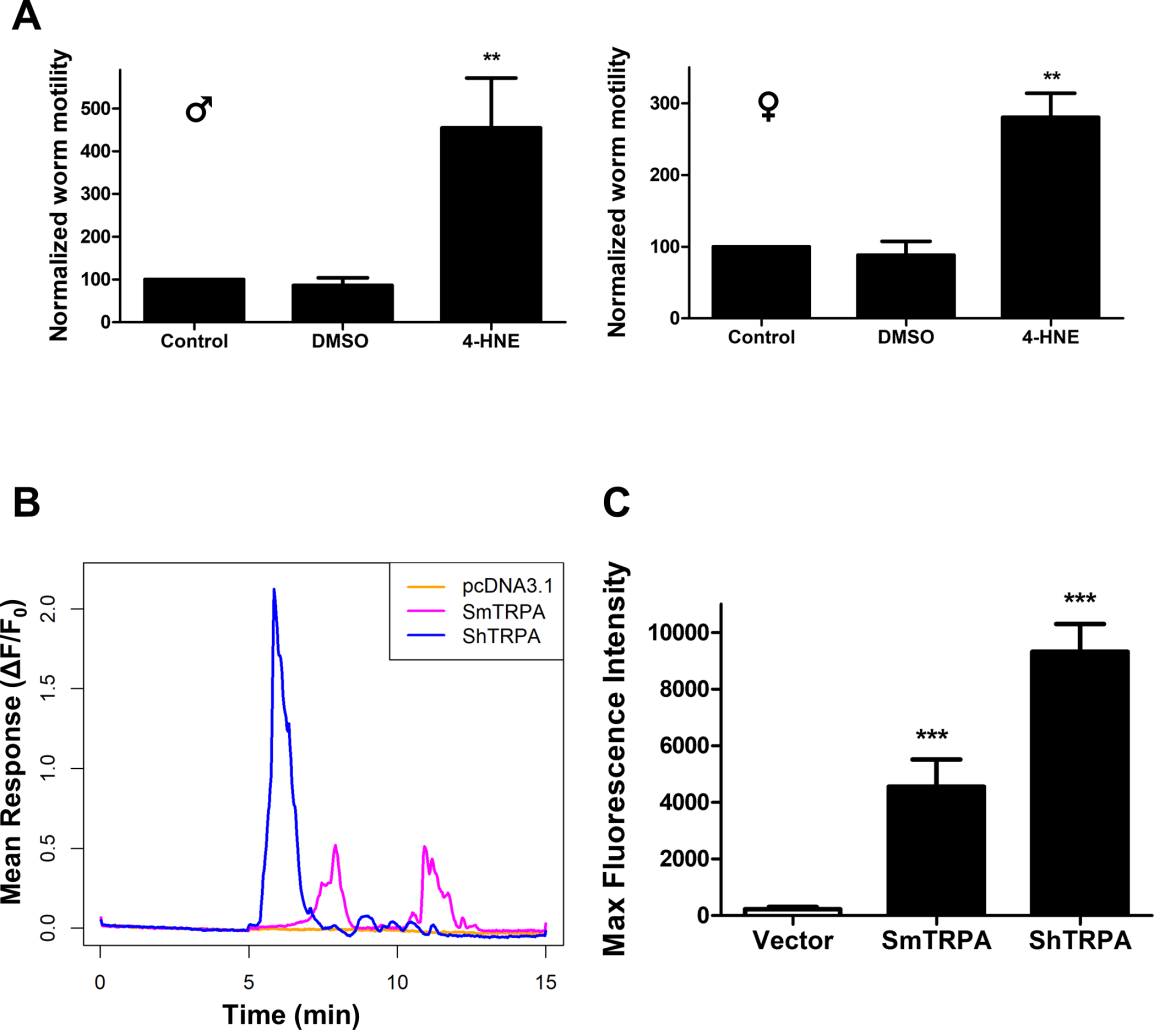
4-HNE, a host-derived, inflammatory activator of mammalian TRPA1, evokes hyperactivity in adult *S*. *mansoni* and activates SmTRPA and ShTRPA. **A.** Response of male (left panel) and female (right panel) *S*. *mansoni* adults to 0.1% DMSO (n = 12) or 10 μM 4-HNE (n = 47, males; n = 24, females), measured as in Fig 2. **, P < 0.01, paired, two-tailed t-test vs. Control. **B.** 4-HNE (50 μM) triggers an increase in intracellular GCaMP6f fluorescence in cells expressing either SmTRPA or ShTRPA. Traces of averaged GCaMP6f fluorescence intensity change in cells transfected with empty vector (pcDNA3.1/zeo^(+)^; orange), SmTRPA (purple), or ShTRPA (blue). **C.** Normalized maximal GCaMP6f fluorescence intensity in response to 50 μM 4-HNE in cells transfected with empty vector (Vector, n = 21, 3 independent transfections); SmTRPA (n = 18, 3 independent transfections); or ShTRPA (n = 95, 4 independent transfections). ***, P < 0.0001, unpaired, two-tailed t-test vs. empty vector data.

Mammalian TRPA1 (and other TRP) channels are modulated by multiple endogenous ligands in mammals [33–37]. For example, 4-HNE, an inflammatory α,β-unsaturated aldehyde produced by lipid peroxidation during oxidative stress, directly activates mammalian TRPA1. To test whether 4-HNE might also influence schistosomes through parasite TRPA1-like channels, we tested the effects of this compound on schistosome locomotor activity and on expressed SmTRPA and ShTRPA. Exposure of *S*. *mansoni* adult male and female worms to 10 μM 4-HNE results in significant parasite hyperactivity (Fig 4A), an effect similar to that elicited by other TRPA1 and TRPV1 agonists [39]. Similarly, 50 μM 4-HNE produces a rise in intracellular Ca^2+^ in CHO cells expressing SmTRPA or ShTRPA (Fig 4B and 4C), indicating that 4-HNE activates schistosome TRPA1-like channels. Interestingly, the response of SmTRPA is smaller, delayed, and shows a second peak, perhaps indicating initiation of an oscillatory 4-HNE-induced Ca^2+^ signal, as is seen in responses to inflammatory and stress signals mediated through TRPA1 in other systems [59]. The response of ShTRPA to 50 μM 4-HNE is neither delayed nor does it exhibit the second peak. These results demonstrate that a host-derived signaling molecule can both impact schistosome motor activity and activate parasite TRPA1-like channels.

## Discussion

Ion channels play key physiological roles in metazoans and are validated targets for several current anthelmintics [15, 16]. However, there is only limited information regarding the expression and function of the majority of ion channel families found in schistosomes and other parasitic helminths. TRP channels, which transduce critical sensory input and regulate other vital functions, represent one largely overlooked helminth channel family. Our previous demonstration that capsaicin and other selective activators of TRPV1 channels produce dramatic changes in motor activity in *S*. *mansoni* adults, schistosomula, and cercariae [39] was a surprising result given that TRPV-like channel genes are not found in the *S*. *mansoni* genome [17, 18]. That this response to capsaicin in adult *S*. *mansoni* was dependent on expression of SmTRPA, a TRPA1-like channel, was also unexpected. Schistosomes also respond to TRPA1 modulators in a SmTRPA-dependent manner, prompting us to ask whether schistosome TRPA1-like channels possess mixed TRPA1/TRPV1-like pharmacology that could account for these types of responses.

The results presented here are consistent with such a mechanism. CHO cells expressing SmTRPA or ShTRPA show a capsaicin-dependent rise in intracellular Ca^2+^. In contrast, and as we have confirmed (Fig 1), TRPA1-like channels from mammals [40, 41] do not display sensitivity to capsaicin (although a recent article [60] reports that 300 μM capsaicin, 30 times higher than the concentration we used, can elicit TRPA1-mediated opening of tight junctions in canine kidney cells). Together with our previous results showing SmTRPA-dependent responses to TRPV1 and TRPA1 activators in whole worms [18, 39], these new findings provide compelling evidence that schistosome TRPA1-like channels exhibit unique pharmacology that may be reflective of functional differences from host channels and that could potentially be exploited for therapeutic targeting.

Interestingly, our experiments also revealed a divergence in the responses of SmTRPA and ShTRPA to AITC, a compound which activates TRPA1 channels in other species through reversible covalent modification of cysteine residues [54, 55]. Application of AITC stimulates an increase in intracellular Ca^2+^ in CHO cells expressing SmTRPA and evokes hyperactivity in *S*. *mansoni* adults [39]. In contrast (Fig 3), AITC has no effect on Ca^2+^ levels in cells expressing ShTRPA and does not stimulate motor activity in adult *S*. *haematobium* females. Although extensive in-depth experiments would be required to understand the basis for this difference, it is notable that ShTRPA appears to lack a cysteine residue (equivalent to rat TRPA1 642 and SmTRPA C631) that has been implicated [54, 55] in the activity of AITC and other electrophilic compounds on TRPA1 channels (S5 Fig). Regardless of the underlying mechanism, if, as in other organisms, TRPA1-like channels transduce sensory signals in schistosomes, this difference in AITC responsiveness could signify divergent TRPA1 channel-mediated responses to host cues in these two species (which migrate to and occupy distinct host predilection sites).

A key question raised by our findings is whether worms produce ligands for these channels, how and under what conditions their production is initiated, and what purpose they may serve. Our finding that 4-HNE acts as an agonist for both SmTRPA and ShTRPA raises the intriguing possibility that host inflammation and oxidative stress are also triggers for parasite channel activation. 4-HNE is a highly bioactive endogenous pro-inflammatory carbonyl species generated through peroxidation of membrane phospholipids in response to tissue injury, inflammation, and oxidative stress. It directly activates mammalian TRPA1 channels [38], providing one of several links between these channels and pro-inflammatory and nociceptive signaling [35, 37]. Here we establish 4-HNE as an endogenous host ligand that can activate schistosome TRPA1-like channels. 4-HNE also increases adult worm motility, though whether that reaction *ex vivo* mirrors the authentic response within the host to 4-HNE is not clear, particularly since higher levels of 4-HNE are required for expressed channel activation (50 μM) than for induction of hyperactivity in whole worms in culture (10 μM). Due to its high reactivity, 4-HNE is known to target several different signaling processes in mammals [61, 62], and it is likely that it also has multiple targets in schistosomes. Notably, however, the concentration of 4-HNE that activates SmTRPA (50 μM) is similar to that required for activation of the mammalian TRPA1 channel and triggering of its linked nociceptive and inflammatory effects [38]. Exploitation and regulation of host inflammatory responses is thought to be a key developmental requirement of schistosomes [63]. Our results using 4-HNE identify a novel mechanism by which parasites might interact with their hosts and pave the way for additional studies to define the functional significance of this interaction. The possibility that schistosome TRP channels, and specifically schistosome TRPA1-like channels, are transducing host signals that are required or exploited by the parasite could present an opportunity to interfere with those interactions, thereby disrupting schistosome development, reproduction, or survival within the host.

## Supporting information

S1 Fig Application of 0.01% DMSO does not increase GCaMP6f fluorescence in CHO cells expressing SmTRPA or ShTRPA. Normalized maximal GCaMP6f fluorescence is shown for cells transfected with pcDNA3.1/zeo^(+)^ (Vector), SmTRPA, or ShTRPA. *, p < 0.05, t-test vs. Vector.
(TIF)

S2 Fig Extracellular Ca^2+^ is required for 10 μM capsaicin to evoke an increase in intracellular GCaMP6f fluorescence in cells expressing SmTRPA. *A*) Traces of averaged GCaMP6f fluorescence intensity change in cells transfected with SmTRPA and exposed to 10 μM capsaicin in our standard solution (Plus Ca^2+^, purple) or in a zero-Ca^2+^ solution (Minus Ca^2+^, brown). *B*) Normalized maximal GCaMP6f fluorescence intensity in response to 10 μM capsaicin in cells transfected with SmTRPA, plus/minus Ca^2+^.
(TIF)

S3 Fig Serotonin and PZQ controls appropriately increase (serotonin) and decrease (PZQ) worm motility. Compounds were applied to worms and motility measured as described in the text. *, P < 0.05, **, P < 0.01, ***, P <0.0001, paired t-test vs. Control, prior to normalization.
(TIF)

S4 Fig Response of rat TRPA1 and TRPV1 channels to AITC. Shown is normalized maximal GCaMP6f fluorescence intensity in response to cells expressing rat TRPV1 (n = 144) or rat TRPA1 (n = 55). ***, P < 0.0001, t-test vs. Vector.
(TIF)

S5 Fig Alignment of a portion of ratTRPV1, SmTRPA, and ShTRPA. Note that one cysteine residue implicated in AITC activity is conserved in all three sequences (denoted by ✓), while another is present in SmTRPA, but absent from ShTRPA (denoted by X).
(TIF)
