# Supplementary figures and images for: Atypical pharmacology of schistosome TRPA1-like ion channels
Source: PLoS Negl Trop Dis. 2018 May 10;12(5):e0006495. doi: 10.1371/journal.pntd.0006495 (PMC5963811; doi:10.1371/journal.pntd.0006495)

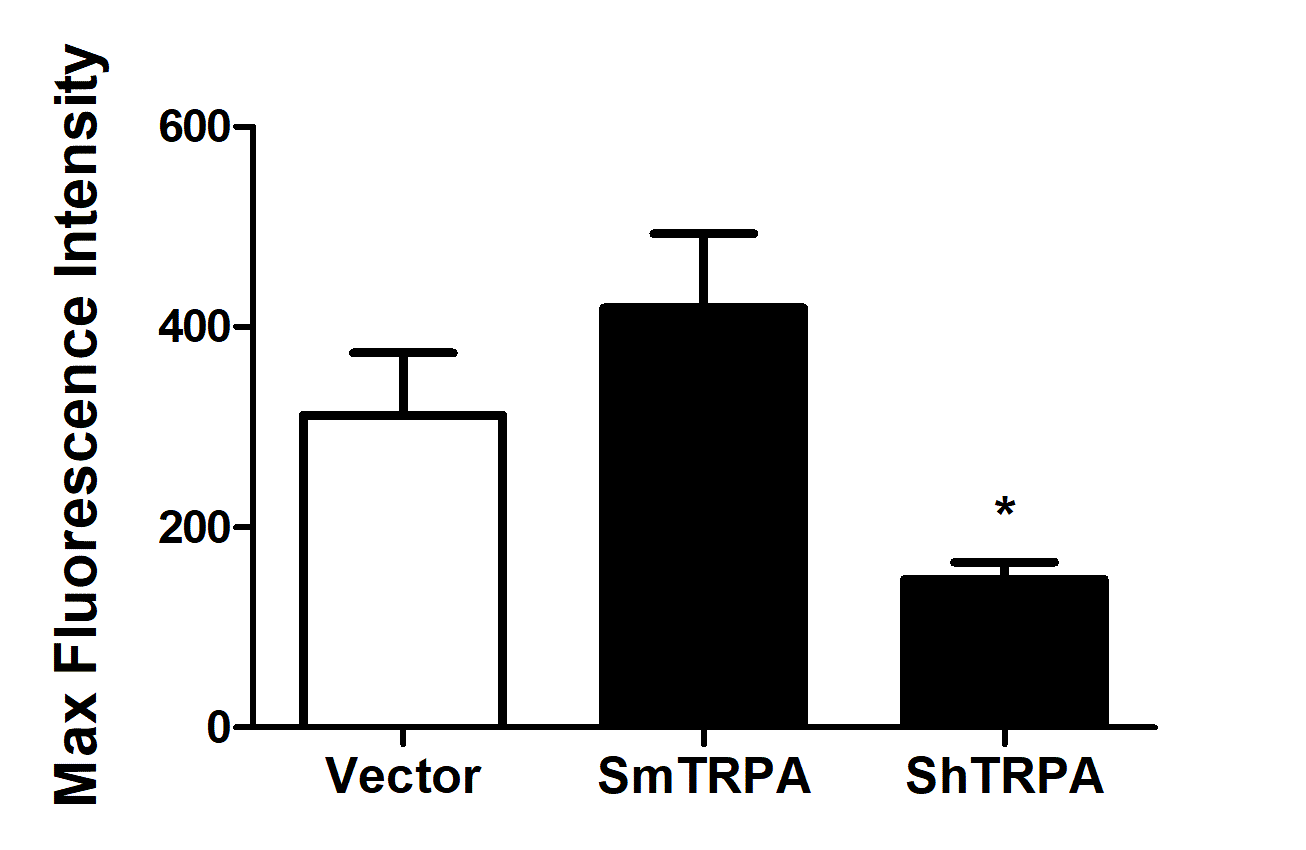

Supplement: S1 Fig — Normalized maximal GCaMP6f fluorescence is shown for cells transfected with pcDNA3.1/zeo(+) (Vector), SmTRPA, or ShTRPA. *, p < 0.05, t-test vs. Vector. (TIF) [file pntd.0006495.s001.tif]

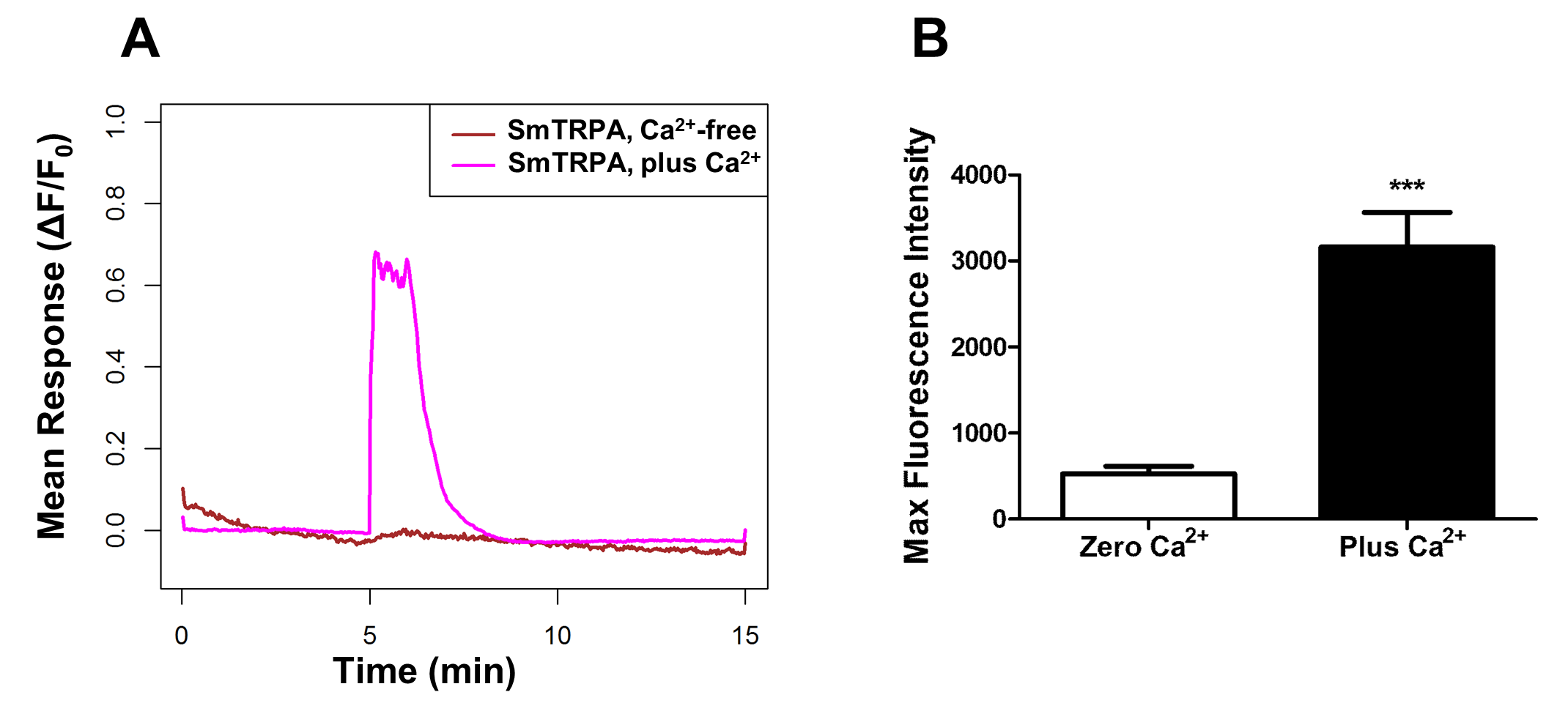

Supplement: S2 Fig — A) Traces of averaged GCaMP6f fluorescence intensity change in cells transfected with SmTRPA and exposed to 10 μM capsaicin in our standard solution (Plus Ca2+, purple) or in a zero-Ca2+ solution (Minus Ca2+, brown). B) Normalized maximal GCaMP6f fluorescence intensity in response to 10 μM capsaicin in cells transfected with SmTRPA, plus/minus Ca2+. (TIF) [file pntd.0006495.s002.tif]

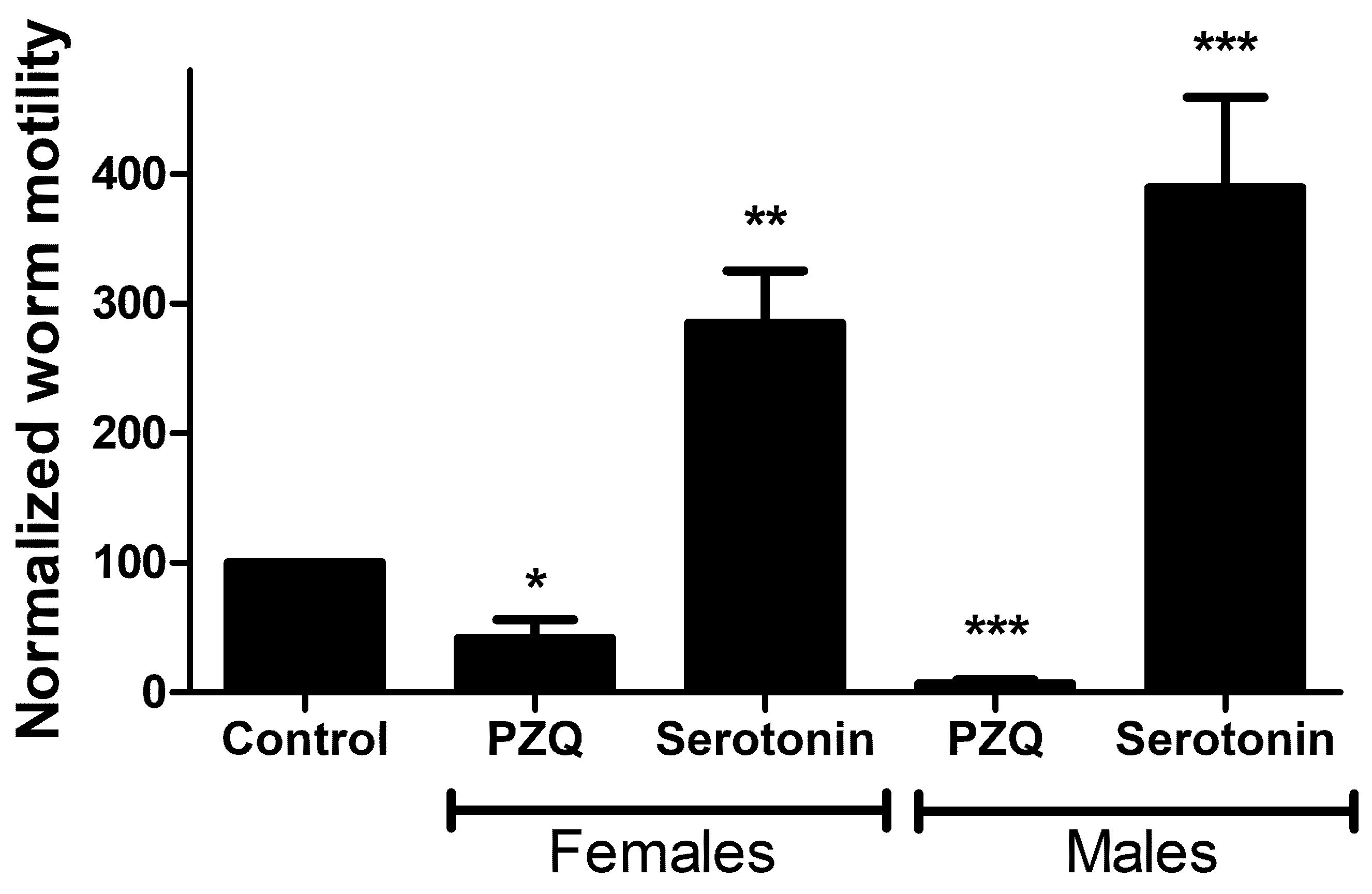

Supplement: S3 Fig — Compounds were applied to worms and motility measured as described in the text. *, P < 0.05, **, P < 0.01, ***, P <0.0001, paired t-test vs. Control, prior to normalization. (TIF) [file pntd.0006495.s003.tif]

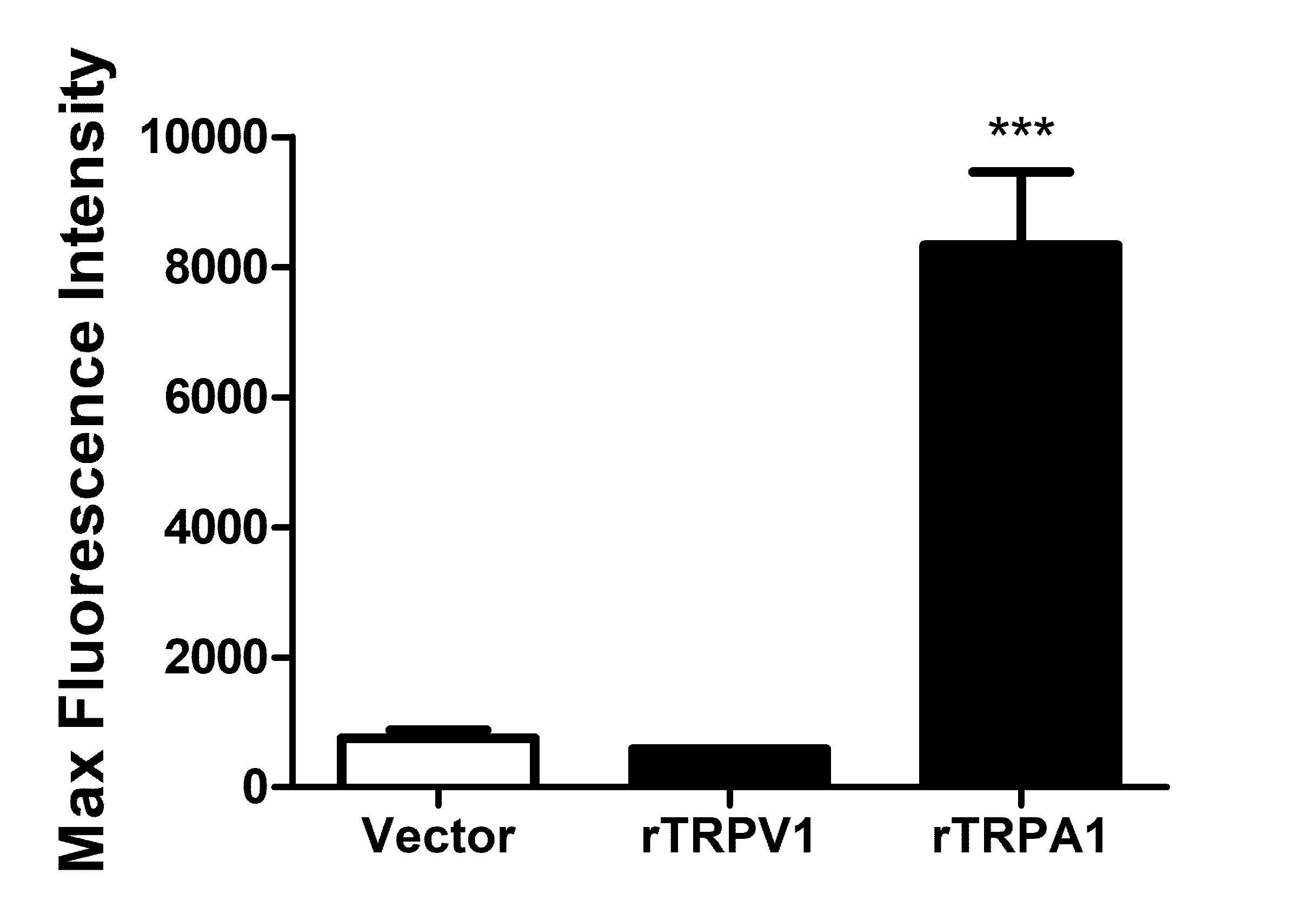

Supplement: S4 Fig — Shown is normalized maximal GCaMP6f fluorescence intensity in response to cells expressing rat TRPV1 (n = 144) or rat TRPA1 (n = 55). ***, P < 0.0001, t-test vs. Vector. (TIF) [file pntd.0006495.s004.tif]

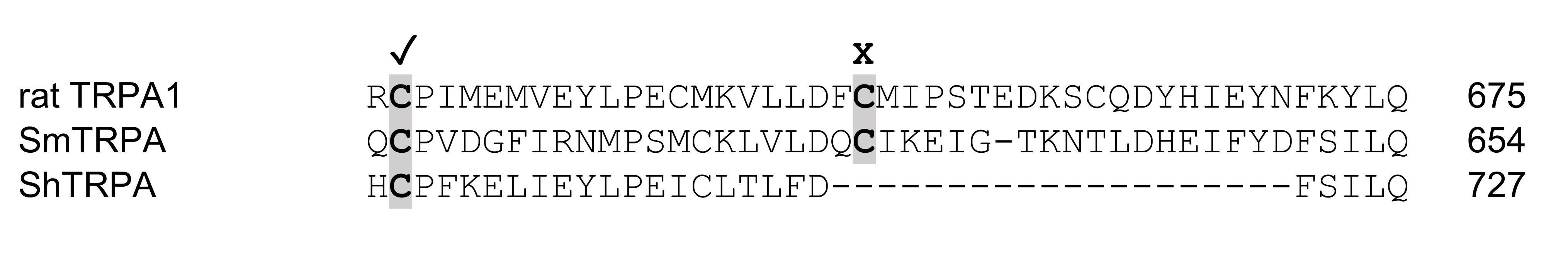

Supplement: S5 Fig — Note that one cysteine residue implicated in AITC activity is conserved in all three sequences (denoted by ✓), while another is present in SmTRPA, but absent from ShTRPA (denoted by X). (TIF) [file pntd.0006495.s005.tif]
